# Entropy-Enhanced Attention Model for Explanation Recommendation

**DOI:** 10.3390/e24040535

**Published:** 2022-04-11

**Authors:** Yongjie Yan, Guang Yu, Xiangbin Yan

**Affiliations:** 1 School of Management, Harbin Institute of Technology, Harbin 150001, China; yanyongjie@hit.edu.cn; 2 School of Mathematics and Computer Science, Jiangxi Science and Technology Normal University, Nanchang 330038, China; 3 School of Economics and Management, University of Science and Technology Beijing, Beijing 100083, China; xbyan@ustb.edu.cn

**Keywords:** entropy, recommendation system, attention mechanism, nerual networks

## Abstract

Most of the existing recommendation systems using deep learning are based on the method of RNN (Recurrent Neural Network). However, due to some inherent defects of RNN, recommendation systems based on RNN are not only very time consuming but also unable to capture the long-range dependencies between user comments. Through the sentiment analysis of user comments, we can better capture the characteristics of user interest. Information entropy can reduce the adverse impact of noise words on the construction of user interests. Information entropy is used to analyze the user information content and filter out users with low information entropy to achieve the purpose of filtering noise data. A self-attention recommendation model based on entropy regularization is proposed to analyze the emotional polarity of the data set. Specifically, to model the mixed interactions from user comments, a multi-head self-attention network is introduced. The loss function of the model is used to realize the interpretability of recommendation systems. The experiment results show that our model outperforms the baseline methods in terms of MAP (Mean Average Precision) and NDCG (Normalized Discounted Cumulative Gain) on several datasets, and it achieves good interpretability.

## 1. Introduction

With the rapid development of the information age, the amount of network data has risen substantially, and the problem of information overload has become increasingly serious. Therefore, in order to provide users with the required information in a timely and efficient manner, recommendation systems [1,2,3,4,5] have emerged as one of the concerns of researchers. Recommendation systems can predict the potential interest according to the differences and preferences of users so as to form a personalized recommendation list. Recommendation systems can effectively help users retrieve information resources that meet their needs in a personalized way and alleviate the problem of information overload. It has been widely used in many fields such as e-commerce [6], social networks [7,8], and so on [9].

The basic principle of the recommendation algorithm based on deep learning usually follows two steps. First, obtain the user’s implicit and explicit information and a series of auxiliary information data such as click-through rate, image content, text content, and browsing duration; take them as the input of the algorithm; and then learn the hidden feature representation of the input data through deep learning technologies such as loop, convolution neural network, self encoder and other deep network models and attention mechanisms. Then these hidden feature representations are calculated by a series of methods, such as softmax function or inner product, to obtain the final prediction or recommendation results. Deep neural networks usually have more layers of hidden layers [10]. A single layer of a hidden layer can abstract the characteristics of input data to another dimensional space and show its more abstract characteristics. Multiple hidden layers can abstract the input features at multiple levels and finally help the model to better linearly divide different types of data in recommendation systems [11]. Because of its learning ability of higher-level and more abstract features, more and more work began to try to use deep neural networks for recommendation tasks. This paper [12] gives a good overview of this aspect.

Recommendation systems can discover the user-personalized interests and unique characteristics of items according to the user historical consumption behavior of items, and they can recommend products or services that users may be interested in. However, there are some problems in these recommendation systems based on interactive records. Recommendation systems can only recommend a product that users are interested in but cannot accurately capture the user points of interest. In other words, recommendation systems cannot clearly tell users why they recommend this product to them; that is, it is not interpretable.

In order to solve the problem of convergence of recommendation lists, diversity is measured with an entropy regularizer to improve the diversity of recommendation lists. We use the user information entropy to express the user’s score distribution and determine the degree of scoring tendency. When determining the nearest neighbor of a user in a traditional user-based collaborative filtering algorithm, we use the information entropy to eliminate some users with obvious different tendencies, which improves the recommendation accuracy. This paper presents a quantitative evaluation method of interpretability of recommendation systems based on text attention mechanisms, which can be used in comment-based in-depth recommendation systems with attention mechanisms. This paper makes a quantitative evaluation score of the interpretability of recommendation systems by judging whether the built-in attention mechanism can really capture the user preference or product feature information reflected in the target comments.

The main contributions of this paper are as follows:

(1) A deep multi-head attention network model based on entropy is proposed. The model considers both review text and goods items, and it uses an attention mechanism to capture the semantic relationship between review content and goods items;
(2) An attention mixing mechanism is designed to construct the review text representation for specific goods items according to the context semantic information;
(3) The effectiveness of the method is verified by simulation experiments. Experimental results show that the collaborative filtering recommendation algorithm based on entropy feature representation can improve the accuracy and interpretability of the recommendation.

## 2. Related Works

### 2.1. Comment-Based Recommendation System

Text information such as user comments and product feature descriptions are common auxiliary information in recommendation systems. The use of text information can alleviate the inherent limitations of recommendation systems to a certain extent. The comments of commodities with high score evaluation will contain more positive commodity characteristics. Therefore, repeated high score comments are used to construct score-enhanced text. Furthermore, the topic features of items are extracted from the comment text based on score enhancement. In the early work, comment-based recommendation systems mainly used topic models to learn potential semantic topics for users and items from comments. Late Dirichlet Allocation (LDA) [13] was used to speculate on the potential topics in the text for recommendation [14]. The core idea of the Latent Factor Model (LFM) [15] is to contact users’ interests and items through implicit features, so as to improve the accuracy of the prediction score and alleviate the sparsity of data. Furthermore, two independent factor learning models are used to mine the common emotional consistency and text consistency of users and commodities, and then, the two models are combined to predict the score. Although the above methods can use the information in the comment text, these methods are based on the word bag model, ignore the word order information and local semantic information, and lose the valuable information in the sentence.

The recommendation model based on deep learning has gradually become the focus of recommendation system research [16,17]. Collaborative Deep Learning [18] uses stacked denoising autoencoders (Sade) to learn the potential features in the text and inputs them into Probabilistic Matrix Factorization (PMF) [19] to obtain the potential matrix of users and items. Compared with the traditional recommendation model, recommendation systems based on deep learning inject nonlinear factors into the model through the nonlinear activation function. It is possible to capture complex interaction patterns between users and items in item interaction records. At the same time, the use of a deep neural network for representation learning can greatly reduce the workload of manually constructing input features, and the network with graph structure and circular structure can also be used to model user items effectively.

### 2.2. Recommendation Method Based on Attention Mechanism

An attention mechanism makes the model focus on important areas and reduces the negative impact of noise so as to improve the recognition performance. In particular, the attention mechanism realizes the element importance evaluation by assigning correlation scores to the elements in the group, and it highlights the element information most related to the task. In addition, it reflects the structural information within the feature set to a certain extent, which is conducive to improving the interpretability of the model.

Attention mechanisms [20,21,22] have been widely used in image processing, machine translation, natural language processing, and other fields. For example, He et al. [23] propose a model that improves an attention network by smoothing users’ historical behavior by discovering the connectivity between items. The model mainly takes advantage of the characteristics whereby user preferences have different priorities. Liu et al. [24] introduce a mechanism to give priority to users’ recent interaction behavior. That is, the model gives priority to the current preferences generated by users’ recent interaction behavior. In order to overcome some inherent problems of RNN, Vaswani et al. [25] proposed a new network structure based only on a self-attention mechanism (SA). There is no need to use a circular mechanism or convolution mechanism at all. Recent related studies [26,27,28] show that a self-attention network can achieve better recommendation results on a variety of recommendation tasks. For example, Zhang et al. [26] propose a method based on self-attention and metric embedding. This method considers both the short-term and long-term preferences of users when solving the sequence recommendation problem. Zhou et al. [29] divide user behaviors into heterogeneous behaviors and other behaviors. They map different heterogeneous behaviors into different potential spaces, and these behaviors can interact in a public space. Considering other influencing factors, a self-attention network is used to model all user behaviors.

In recent years, a recommendation method based on a graph neural network and attention mechanism can divide regions for the potential characteristics of items according to user preference differences [30,31]. Lin et al. [32] give high weight to the regions concerned by most users with multi-level attention for recommendation. In essence, its working principle is to use the probability distribution of attention and capture inputs that have a critical impact on the output.

Song et al. [33] have integrated the attention mechanism and a deep learning model, which has promoted the development of recommendation systems. Ren et al. [34] applies the dynamic graph attention mechanism model and RNN model to community recommendation. The literature believes that user preferences are affected by the preferences of friends on social platforms. The graph attention mechanism model can dynamically capture the impact of long-term and short-term preference changes of users’ friends on users.

The model proposed in the literature makes full use of users’ social relations and captures the preferences of users’ friends. However, the feature extraction of users and friends is not accurate enough, and the long-term dependence between users and recommended items is not considered.

In order to solve the problem of timing data of microblog topic tags, Li et al. [35] construct an LSTM model based on topic attention mechanism. The model takes into account the time factor, integrates the timing characteristics into the model, and effectively improves the performance of recommendation. However, the model does not consider the impact of user information and microblog tag text length on recommendation results. To solve this problem, Sun et al. [36] proposed a temporal enhanced statement set LSTM model based on an attention model. The model analyzes and describes the microblog features from the word level and statement level, and it fuses the time information in the statement set attention level, which fully reduces the impact of noise data on the classifier in the microblog tag data. Therefore, in addition to solving the problem of microblog topic tag recommendation, the model can also be used to solve the problems of text recognition, language translation, and dynamic recommendation. However, the LSTM model can only deal with single Euclidean spatial data, and it cannot deal with more complex non-Euclidean spatial data.

The recommendation model of dual attention network learning with dual social effects is proposed in [37]. The dual attention mechanism of the model includes two aspects: modeling according to the attention weight assigned by the user and dynamic attention modeling through context awareness. Through dual modeling, the user’s social effects are effectively transmitted to the field of recommended items. It alleviates the problem of data sparsity often encountered in traditional recommendation systems. Sun et al. [38] proposed a sequence recommendation model called BERT4Rec, which uses deep two-way self-attention to model the sequence of user behavior. In this way, the system learns the two-way representation model, which allows each item in the user’s historical behavior to fuse the information on the left and right sides to make recommendations.

### 2.3. Convolutional Neural Network Based on Attention Mechanism

In 2016, Gong et al. [39] proposed a CNN Sina Weibo topic recommendation model based on an attention mechanism. The model set up two attention channels (global and local) to improve the accuracy of recommendation. However, the data used in the model are text types, ignoring other forms of topic types such as images. In order to solve this problem, Zhang et al. [40] proposed a collaborative attention mechanism model, which fully considered the dependency between text, image and microblog topic tag. Due to sufficient factors, its recommendation performance is better than that considering only text. A deep cooperative neural networks-based on attention (ACONN) model was proposed. The function of the attention mechanism is to reassign the weight of the text matrix. The function of the parallel CNN model is to fully mine the information of users and texts to obtain potential hidden features. Compared with other deep learning models, this model has the advantages of less parameters and lower complexity. The model can learn the potential hidden features of target users so as to improve the effect of recommendation. It makes full use of the characteristic whereby an attention mechanism can capture information with large weight, and the advantages of the CNN model in weight sharing and local connection. Although it improves the effect of recommendation, the problem of data sparsity will gradually appear when the data scale is large enough. The RNN model combined with an attention mechanism [41] has greatly improved the extraction efficiency of key features when extracting features. Therefore, the accuracy of recommended items has also been greatly improved. However, the model cannot recommend users’ dynamic preferences in real time.

A neural news recommendation model based on personalized attention (NPA) is proposed by Wu et al. [42]. The core of this method is a news representation model and user representation model. In the news representation model, the authors use a convolutional neural network to learn the implicit representation of news articles based on headlines. A large number of experiments are carried out on the real news recommendation data set collected in MSN news, and the results show the effectiveness of the methods proposed by the authors in news recommendation. Cross-domain recommendation is an important method to solve the problem of data sparsity. The auxiliary information in multiple domains can serve the recommendation in the target domain by inputting auxiliary information. The model can learn the potential hidden features of target users so as to improve the effect of recommendation.

### 2.4. Recommendation Method Based on Interpretability

Interpretability provides a reasonable explanation for the decision making of recommendation systems [43], which can effectively improve the transparency, persuasion, and credibility of recommendation systems as well as the user experience. Li et al. [44] introduce a method that can connect ID and text and endow ID with linguistic meaning, which solves the problem in which it is difficult for the transformer to use an ID to generate personalized text. User comments refer to the user textual feedback on the items and services after purchasing items or receiving services. Xie et al. [45] utilize an interpretable recommendation framework based on a knowledge graph and multi-objective optimization, which can optimize the accuracy, diversity, and interpretability of recommendation at the same time. The comments contain rich information about the user personality preferences and commodity characteristics, such as the description of commodity performance (such as commodity specification and quality) or some obvious emotional tendencies, which provides data support for recommendation systems to better extract user information. Therefore, the comment-based recommendation system can effectively improve the accuracy and interpretability of recommendation [46].

## 3. Preliminaries

In this section, we introduce the concepts of the attention mechanism with entropy and formulate the problem. Some important notations are summarized in Table 1.

### 3.1. Entropy

Shannon information entropy [47] describes the probability distribution characteristics of variables, so the distribution characteristics of scoring values such as user rating score concentration, richness, and extremes can be mined. The closer the scoring information entropy between users, the more consistent the scoring distribution characteristics and the greater the similarity of users. Entropy is introduced into recommendation systems’ objective function as a regularization term.

Let α be a discrete random variable in *A*, with a finite range $a_{1},\ldots ,a_{N}$. Let $p_{i}$ be the probability of the event $\alpha =a_{i}$. Then, the Shannon entropy of *A* is denoted as H(A)

(1) $$H\left(A\right)=\sum_{i}p_{i}\log_{2}\frac{1}{p_{i}}=-\sum_{i}p_{i}\log_{2}p_{i}$$

Let f(σ) be the relative frequency of symbol σ in *A*. Then, its entropy is

(2) $$H\left(A\right)=-\sum_{\sigma \in \Sigma }f\left(\sigma \right)\log_{2}f\left(\sigma \right)$$

### 3.2. Transformer

Vaswani [25] used transformer architecture instead of seq2seq and self-attention instead of LSTM and achieved better results in translation and other tasks. The transformer model uses multi-head attention in the encoder and decoder based on a self-attention network, respectively. The attention layer connecting the encoder and decoder is an important part of the transformer model.

The attention formula used is the following:

(3) $$\mathrm{Attention}\left(Q,K,V\right)=softmax\left(\frac{QK^{T}}{\sqrt{d_{k}}}\right)V$$

The softmax funtion is given by $\sigma {\left(z\right)}_{i}=\frac{e^{z_{i}}}{\sum_{j=1}^{L}e^{z_{j}}}$, for i = 1, *…*, K and z = $\left(z_{1},\ldots ,z_{K}\right)\in R^{K}$.

For each query, the attention score of each value is the dot product between the query and the corresponding key. The score of value is divided by $\sqrt{d_{k}}$ to prevent huge values.

Instead of performing a single attention function with $d_{model}$ dimensional keys, values, and queries, researchers found it beneficial to linearly indicate the queries, keys, and values h=8 times with different, learned linear items to $d_{q}$, $d_{k}$, and $d_{v}$ dimensions, respectively. On each of the versions of queries, keys, and values, we then perform the attention function in parallel, yielding $d_{v}$-dimensional output values. Multi-head attention allows the model to jointly attend to information from different subspaces at different positions. Then, the multi-head formula is the following:

(4) $$\mathrm{MultiHead}\left(Q,K,V\right)=Concat\left(head_{1},...,head_{h}\right)W_{n}^{O}$$

where $head_{i}=attention\left(QW_{i}^{Q},KW_{i}^{K},VW_{i}^{V}\right)$, the items are parameters matrices $W_{i}^{Q}\in R^{d_{model}\times d_{q}}$, $W_{i}^{K}\in R^{d_{model}\times d_{k}}$, $W_{i}^{V}\in R^{d_{model}\times d_{v}}$, and $W_{n}^{O}\in R^{d_{h}\times d_{h}}$.

## 4. Our Model

The relationship between different aspects mentioned in the same sentence is modeled through additional attention layers. In addition, our model analyzes the relationship between words through entropy, introduces the global sentence representation into the existing attention mechanism, and designs additional auxiliary tasks to guide sentence learning. This paper also introduces location information and part of speech information to increase the selection ability of the model so as to realize the prediction of emotional polarity.

### 4.1. Sentiment Analysis Based on Entropy

There are some recommendation systems used such as fuzzy entropy [48], relative entropy [49] and maximum entropy [50]. It can be seen from Equation (1) that the information entropy H(x) is only related to the probability distribution of variable *x* but has nothing to do with its specific value. To some extent, this shows that information entropy can effectively avoid the interference of noise data and effectively filter out users with less scoring information in the scoring system. Users in the system have different effects on the recommendation engine. Some users provide more information in the score, while others contain less information. Therefore, effectively filtering users with less information can effectively improve the recommendation accuracy.

In order to introduce the user information entropy model into recommendation systems, for user *u*, the score set is represented by $R_{u}=R_{1},R_{2},\ldots ,R_{n},\ldots ,R_{s}$. In the scoring system with a score of 1 to 3, respectively, corresponding to positive, negative, and neutral, $R_{n}\in 1,2,3$, where p=∥Ru∥ represents the score generated by user *u* in the system. For user *u*, according to Equation (1), the information entropy is:

(5) $$H\left(U\right)=-\sum_{i=1}^{C}p_{uk}\log_{2}p_{uk}$$

where *C* represents the number of scoring intervals, and in the three-point scoring system, C=3; $P_{uk}$ is the probability that user u’s score falls within interval K. The calculation process of $P_{uk}$ is as follows:

(6) $$P_{uk}=\frac{\left[\sum_{R_{n}\in R_{u}}Ir_{n}=k\right]}{∥R_{u}∥}$$

where k∈1,2,3, I{∗} is the indicator function, I{true}=1, I{false}=0. Combined equations refeq:entropy and (6) can calculate the information entropy according to the user’s score value. From the perspective of information theory, according to the characteristics of centralized scoring and extreme scoring of naval users or a small number of normal users who produce noise data, this paper directly uses information entropy to measure the amount of information contained in users’ scoring and filters users with low information entropy to achieve the purpose of filtering noise data. For example, in Table 2 with a score of 1 to 3, if user *u* evaluates 15 items, and there are 6 items from 1 to 3, then its information entropy $H\left(U\right)=-\sum_{1}^{3}\frac{6}{15}\log_{2}\frac{6}{15}\approx 1.32$, and its information entropy reaches the maximum. Because its scores are evenly distributed, it can indicate that it is more cautious and objective in scoring the corresponding items. In another extreme case, the user u scores all items with 1, that is, $p_{u1}=1$, which can be calculated by substituting into the formula to get H(U)=0. Therefore, the user’s information entropy reaches the lowest value, which belongs to noise data. Intuitively, it can also be seen that the user’s scoring behavior is too arbitrary and extreme, and the reliability is low.

After processing, a new scoring matrix $R_{new}$ is obtained from the original scoring matrix *R*. Obviously, $R_{new}$ has higher data quality, and the collaborative filtering model based on $R_{new}$ training will also have higher recommendation accuracy.

### 4.2. Model Architecture

**Figure 1:**
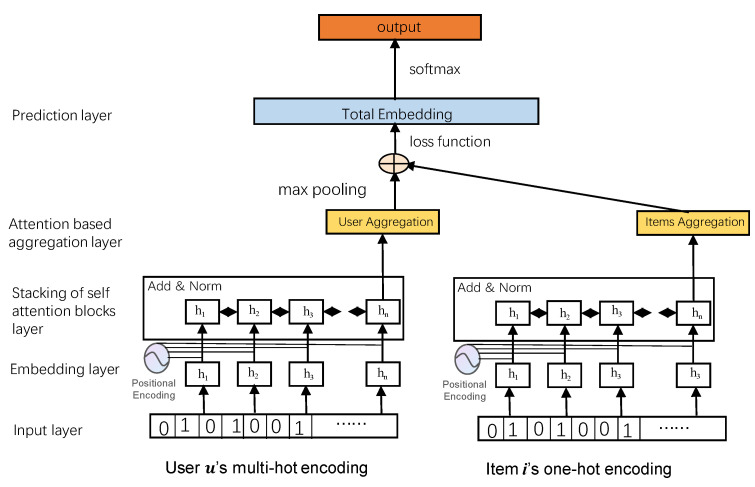
Architecture of our model.

When the interactive sequence is given non-uniform weights, the complexity of the model can be reduced, and the long-term information of the sequence can be captured more concisely. In order to achieve this effect, that is, to suppress the tendency of uniform distribution of attention weight, reduce the number of captured actions, and improve the ability to distinguish items, considering that the attention weight is given adaptively by the model, try to increase the entropy-positive term of attention weight on the original loss function to form a new recommendation model structure, as shown in Figure 1. The main components of the model are described layer by layer from bottom to top.

(1) Input layer

The bottom layer of our model is the input layer, which is divided into three parts: the input of historical interactive items, the input of target items, and the input of short-term sequential interactive items. The input of historical interactive items is represented by the ID multi-hot encoding of these items. The input of the target item is represented by one-hot encoding the ID of the item. The input of short-term sequential interactive items is represented by multiple thermal codes for the ID of the item. The final result of the input layer is the encoded feature vector.

(2) Embedding layer

The input layer is followed by the embedding layer, which is a fully connected layer. First, the original sequence is processed, and then, the processed sequence is input into the embedding layer to obtain the embedding vector. The model can only deal with the sequence with a fixed length *n*, which is the maximum length of the training sequence. If the length of the original sequence is less than *n*, fill 0 from the left. If the length of the original sequence is greater than *n*, the sequence with the nearest length is intercepted. In this way, the input sequence $\left(S_{1}^{u},S_{2}^{u},S_{3}^{u},\ldots ,S_{|S^{u}|-1}^{u}\right)$ is transformed into a sequence of fixed length sequence $s=(s_{1},s_{2},s_{3},\ldots ,s_{n})$. If the sequence length is greater than *n*, take the *n* recent actions. If the sequence length is less than *n*, repeatedly add a ‘padding item’ to the left. The sparse vector can be embedded into a linear matrix, which makes the sparse vector have the corresponding meaning. This feature is very suitable for deep learning. Especially in the recommendation field, the recommendation sequence will be determined according to the calculated embedding similarity between users and items or between items.

The sparse feature vector obtained by the input layer is transformed into a low-dimensional dense implicit vector representation in the implicit space. The sequence is embedded into the matrix $E_{i}=M_{s_{i}}$ through items *M*, which represents the collection of all items and the embedding dimension. In the sequential recommendation task, items have a strict sequence, so the location information needs to be embedded. The self-attention mechanism cannot perceive the location information; that is, the position of elements in the exchange sequence does not affect the final result. This behavior of not distinguishing the chronological order is contrary to the serialization recommendation. Therefore, position embedding $P\in R^{n\times d}$ is added in the above embedding vector *E* to obtain the input embedding with position information $\hat{E}$. Equation (7) gives a detailed definition.

(7) $$\hat{E}=\left[\begin{array}{c} M_{s_{1}}+P_{1}\\ M_{s_{2}}+P_{2}\\ ...\\ M_{s_{n}}+P_{n} \end{array}\right]$$

(3) Stacking of self-attention blocks

This module is composed of one or more self-attention blocks stacked, and each self-attention block is composed of a self-attention layer, feed-forward network, residual connection, normalization layer, and dropout layer.

Self-attention layer: In Transformer Equation (3), the attention mechanism function of the scaling dot product is defined. In fact, the attention function is used to calculate the degree of correlation between *Q* and *K*, distribute the weight according to the degree of correlation, and calculate the weighted sum of *V*. The input embedded $\hat{E}$ converts into three matrices $W_{Q},W_{K},W_{V}$ and then inputs the self-attention function:

(8) $$S=SA\left(\hat{E}\right)=\mathrm{Attention}\left(\hat{E}Q,\hat{E}K,\hat{E}V\right)=softmax\left(\frac{\hat{E}Q\hat{E}K^{T}}{\sqrt{d_{k}}}\right)\hat{E}V$$

**Figure 2:**
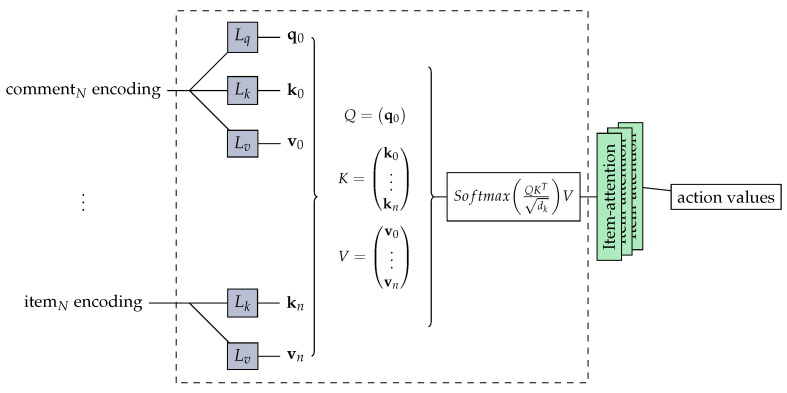
Internal structure of stacking of self-attention blocks. It is composed of a stack of item-attention heads. The blocks $L_{q}$, $L_{k}$, and $L_{v}$ are linear layers. The keys *K* and values *V* are concatenated from all items, while the query *Q* is produced by user comments.

The internal structure of stacking of self-attention blocks is shown in Figure 2.

Feed-forward network: Considering that the self-attention layer is a linear model and cannot perceive the nonlinear interaction of hidden features in different dimensions, it is necessary to use the nonlinear activation function to introduce nonlinear factors, that is, add a two-layer point feed-forward network:

(9) $$FFN\left(S_{i}\right)=ReLU\left(S_{i}W^{\left(1\right)}+b^{\left(1\right)}\right)W^{\left(2\right)}+b^{\left(2\right)}$$

where $W^{\left(1\right)},W^{\left(2\right)}\in R^{d\times d}$, $b^{\left(1\right)},b^{\left(2\right)}\in R^{d}$. The ReLU activation function is nonlinear, which can make neurons have sparse activation, avoids the problem of gradient explosion or disappearance, and has fast convergence speed, which can help the model better mine relevant features.

Stacking of self-attention blocks: Stacking multiple self-attention blocks can make the model learn more complex feature transformation. However, through increasing the number of layers of the network in this way, it is easy to cause problems such as over fitting, gradient disappearance, and training time growth. Therefore, it is necessary to add residual connection, a normalization layer, and a dropout layer. The stacking formula of multiple self-attention blocks is defined as follows:

(10) $$x^{\prime }=LayerNorm\left(x+Dropout\left(g\left(x\right)\right)\right)$$

where *g* represents self-attention layer or feed-forward network. LayerNorm is layer normalization, which is defined as $LayerNorm\left(x\right)=\alpha ⨀\frac{x-\mu }{\sqrt{\sigma^{2}+\epsilon }}+\beta$.

(4) Aggregation layer based on attention mechanism

The number of interactive items of users is not only one, so there are multiple results after the implicit vector inner product operation obtained through the interaction function in the interaction layer. The purpose of the aggregation layer is to aggregate these inner product results. Combined operation is used to facilitate subsequent processing. Our model supports traditional aggregation strategies, such as max pooling.

Considering the different contributions of different items to the prediction, we use the attention-based aggregation strategy. At the same time, the experimental part (Section 5) gives the attention-based aggregation strategy and communication. The experimental results of the unified aggregation strategy verify the effectiveness of the attention-based aggregation layer designed in this paper.

(5) Prediction layer

Using the idea of matrix decomposition, at each time step *t*, the effective information $F_{t}^{\left(b\right)}$ extracted by the model and items $N_{i}$ are embedded into the dot product, the score $r_{i,t}$ is calculated, and then, it is sorted for recommendation.

(11) $$r_{i,t}=F_{t}^{\left(b\right)}N_{i}^{I}$$

where $r_{i,t}$ represents the given interaction sequence $\left(s_{1},s_{2},\ldots ,s_{t}\right)$, and the possibility of the next item predicted by the model is item *i*. $N\in R^{I\times d}$ is a trained item embedding matrix, *I* is the collection of all items, and *d* is the dimension of the embedded vector.

Specifically, an entropy regular term is added to the original binary cross-entropy loss function in Equation (2). At first, the distribution of the attention network is very sparse. Thus, we add an entropy in our loss function to make the distribution more concentrated. We call it an entropy-enhanced attention network. The entropy regular term is calculated from the self-attention matrix in the first self-attention block. The entropy value of each element is added to the loss function to form a new loss function as follows:

(12) $$Loss=-\frac{1}{K}\sum_{i}^{K}p\left(y_{i}\right)\log p(\bar{y_{i}})-\gamma \sum_{m}\sum_{r}p\left(r\right)\log p(r)$$

where *m* denotes audio and visual modalites. *r* represents each distribution in *m*. γ is a hyperparameter, and *K* denotes sentence length.

The following Algorithm 1 shows the detailed flow used in the paper.
**Algorithm 1:** Recommendation based on attention networks with entropy function.**Input:** user dataset *User*, item dataset *Item*,            review dataset *Review*, vocabulary *V*;**Output:** user representation *U*, item representation *I*,               recommendation list *L*;1: Initialize embedding size = 200, batch size = 32, negative sample =5;2:    **for** epoch = 1,2,⋯,n **do**3:       split the dataset *User*, *Item* and *Review* into4:          training datasets (80%), verification datasets (10%), and testing datasets (10%);5:       construct according to Equation (7);6:       learn the FFN according to Equation (9);7:          expected LayerNorm according to Equation (10);8:       get Loss function according to Equation (12);9:    **end for**10: **return** recommendation list *L*.

## 5. Experiments

### 5.1. The Datasets

In this section, we will introduce the data sets used in this paper, including some public data sets, such as restaurant, laptop, and twitter in the SemEval 2014 Task 4 (https://alt.qcri.org/semeval2014/task4/, accessed on 6 December 2021), which is also explained in [51].

Semeval 2014 task 4 is a widely used data set in the field of aspect-based sentiment analysis. Aspect-based affective analysis aims to extract the affective polarity of an aspect or goal in a sentence. This data set consists of manually labeled restaurant and laptop comments. The data set is divided into two parts: a training set and test set. Table 2 shows statistics for restaurant and laptop data sets. It can be seen from the data in the table that the distribution of positive, negative, and neutral tags is uneven in the training set and test set, especially in the laptop laptop comment data set. At the same time, the amount of training data is not very large. These characteristics make this data set more difficult. Both restaurant and laptop data sets are formal data, with complete sentences and standardized syntax.

### 5.2. The Measurements

Two indicators (*MAP@u*, *NDCG@u*) are used to measure the experimental results:

*MAP@u*, Mean Average Precision:

(13) $$MAP@u=\frac{\sum_{u\in U^{te}}AP@u}{|U^{te}|}$$

*NDCG@u*: Normalized Breakage Cumulative Gain is used to calculate the ranking quality of recommended items. The value of the *NDCG@u* is between (0,1]. The NDCG@u for *u* is defined as:

(14) $$NDCG@u=\frac{\sum_{i=1}^{p}\frac{2^{rel_{i}}-1}{\log_{2}\left(i+1\right)}@u}{|U^{te}|}$$

$rel_{i}$ indicates the relevance of the results in position *i*.

### 5.3. The Results

We performed the experiments on a GPU GeForce GTX 1080 Ti with Ubuntu 18.04 operating system. The comparison method and the model proposed are implemented based on a Pytorch framework, and its parameters are obtained by the cross-validation method. In the experiment, 80% of the data set is used as the training set, 10% is used as the verification set and 10% is used as the test set. Five cross experiments are carried out, and the average result is taken as the final result.

Some conflicting words are removed, and all words are converted to lower case. Any stop words, symbols, and numbers are not removed. The word segmentation tool provided by the nltk (http://www.nltk.org/, accessed on 26 February 2022) tool is used to segment all sentences. All sentences are filled with the “pad” character to the maximum length, and the maximum length of the sentence is set to 30. We use the glove [52] word vector to initialize our word vector. The word vector dimension is 300, and the dictionary size is 2 MB (https://nlp.stanford.edu/projects/glove/, accessed on 15 January 2022). The value of the word vector adjusts in the process of training.

The emotional polarity of a sentence is not only determined by the content but also has a strong correlation with the evaluation object. We first evaluate SemEval 2014 Task 4 data sets, whose characteristics are summarized in Table 2. ATAE-LSTM, IAN, BILSTM-ATT-G, MemNet, and TNet model are proved to be successful, and the results of the Top-k recommendation task are improved in previous research work. We use the following five representative start-of-the-art models as the experimental baseline:

- **ATAE-LSTM** In this paper, attention and LSTM are combined to obtain more important context information for different aspects through attention so as to solve the problem of aspect level sentiment analysis, which has achieved good results in the experimental data set [53];
- **IAN** uses interactive locality to calculate the expression of sentence and target by using the attention method and realize the interaction between target and context [54];
- **BILSTM-ATT-G** uses the gate to control the importance of the left and right parts of the target and establish the relationship between target and context [55];
- **MemNet** takes the word vector as a memory unit and uses the multi-layer attention method to obtain the final representation. In order to overcome the disadvantage that the attention mechanism cannot obtain the timing information, it also uses the position weight [56];
- **TNet** proposes to generate sentence representation related to the target, combined with context information [57]. A transformer unit for target representation is proposed so that the target information can be better represented.

The model parameters are set as follows: the number of layers of thetransformer is 12, the number of heads of multi-headed attention is 12, the dimension of the word vector is 768, and the dimension of the middle layer is 3072. We first load the pre-training parameters of OpenAI GPT [58] and then tune them together with the subsequent structure. The result is shown in Table 3.

The experiment uses five public data sets: Amazon beauty and games (http://jmcauley.ucsd.edu/data/amazon/, accessed on 15 January 2022); the Steam data set introduced in [59]; and the Movielens1M and Movielens10M data sets [60] cleaned by us. Comparing the performance of different algorithms, there are millions of types of commodity comment information in the original data set without category filtering and data sorting. First, we conduct data preprocessing to screen users with less than five comment records and commodities with less than five comment records. The evaluation value in the data set is an integer between 1 and 5, and then, we filter the category of the data sets. The statistical information of the data sets after preprocessing is shown in Table 4.

The scores of these data sets are between 1 and 5. We remove 2 and 4 to make the remaining three numbers correspond to three different polarities: 1 corresponds to negative; 3 corresponds to neutral; and 5 corresponds to positive.

The result is shown in Table 5. In all neural network models, **ATAE-LSTM** mconsiders the target and uses the attention method, and the effect is improved. **IAN** uses sentence to target attention and target to sentence attention, which further improves the experimental effect. The experimental results of **BILSTM-ATT-G** and **MemNet** on Amazon games and steam are good, but the effect on the ML-1M and ML-10M data sets is not so much improved. It can be seen that LSTM is not good at dealing with a large number of spoken texts in twitter data. **TNet** uses CNN and LSTM, and it has achieved good results on five data sets. Our model uses transformer to extract sentence features, which makes it easier to deal with long-term dependencies. Our model extracts features from different granularities, and the experimental results on five data sets are better than those on other models.

In this paper, the attention weight of our model is obtained by weighting the positive sentiment weight and negative sentiment weight. The attention weight itself weakens the interpretability of the model to a certain extent. The selected data set contains more nouns and adjectives. In ML-1M dataset, users such as “My Fair Lady (1964)” whose genres are “Musical∣Romance”, are more like “Roman Holiday (1953)” whose genres are “Comedy∣Romance”. The higher the proportion of adjectives with high weight, the stronger the interpretability of the recommendation system. The final recommendation is a mixture of recommendations from different genres, in which each recommendation is interpreted by specific genres.

Our model adds the entropy-enhanced attention mechanism to the influence of context to the attribute words and further obtains the representation of attribute words, which makes the words more accurate. Our model updates the representation of context after updating the representation of attribute words. The resulting design of the entropy-enhanced attention mechanism makes the representation of context more accurate. Compared with the use of the self-attention mechanism, our model improves the accuracy to a certain extent.

In the fusion process of multiple users’ comments and item attributes, we use the attention mechanism with entropy function to fuse a variety of information. Compared with the simple average summation algorithm, our model is more consistent with the different attraction characteristics of type information to users, and different information also plays a different role in the final scoring process of products. Finally, according to the learned attention weight, the corresponding information is selected as the explanation of recommendation.

## 6. Conclusions

We analyze the shortcomings of the existing recommendation methods based on users’ comments. In addition, existing methods have fixed the contribution degree of the same item, but in fact, the contribution of the same item to the prediction will change over time. The above shortcomings limit the recommendation performance of the existing methods to a certain extent.

In view of the above shortcomings, firstly, we design a recommendation model with joint attention mechanisms, which can adaptively model users’ preference by using user’s comments. Specifically, when modeling users’ preference, the attention network is designed to adapt to the attention weight of different historical interactive items to predict the contribution of target items. When modeling users’ preferences, the design of theattention network with information entropy is adapted to learn the attention weight of different interactive items to predict the contribution of target items rather than fixed modeling, so it can also learn the contribution weight of items over time. Finally, we analyze the performance between ours and other models; that is, our model has a general structure.

The experimental results show that our model is superior to other mainstream benchmark models in the two mainstream evaluation indexes (MAP and NDCG), which verifies the effectiveness and rationality of the model. Experiments verify that the entropy function in the attention network is effective and explainable.

In the real scene, in addition to the comments between items, other factors may also have a certain impact on users’ recommendation, such as the geographical location letter and users’ social relationship information. Therefore, in future work, we can consider effectively integrating these factors into our model and further expanding it to improve the recommendation performance.

## Figures and Tables

**Table 1:** Important notations.

Notation	Description
U, u	user entities set U, u∈U
I, i	item entities set I, i∈I
W	weight matrix in attention network
N	the number of recommended items
Q	the number of queries
K	a mapping of sequence of keys
V	the number of value
σ	sigmoid function

**Table 2:** Semeval 2014 task 4 dataset statistics.

Datasets	Positive (Score 3)	Neutral (Score 2)	Negative (Score 1)
Laptop-Train	994	464	870
Laptop-Test	341	169	128
Restaurant-Train	2164	637	807
Restaurant-Test	728	196	196

**Table 3:** The experimental results of using information entropy to evaluate the polarity of the data sets where u=30. The highest value is in bold.

Models	Restaurant		Laptop	
	MAP	NDCG	MAP	NDCG
ATAE-LSTM	0.435	0.339	0.352	0.512
IAN	0.425	0.342	0.361	0.526
BILSTM-ATT-G	0.439	0.328	0.348	0.485
MemNet	0.441	0.340	0.357	0.463
TNet	0.436	0.345	0.346	0.491
Ours	**0.452**	**0.355**	**0.415**	**0.562**

**Table 4:** Statistics of data sets.

Dataset	# Users	# Items	# Avg Sequence len	# Max Sequence len
Amazon beauty	52,024	57,289	7.6	291
Amazon games	31,013	23,715	7.3	858
Steam	334,730	13,047	11.0	1229
ML-1M	6040	3416	163.5	2275
ML-10M	69,878	65,133	141.1	7357

**Table 5:** The experimental results of the Table 4 data sets, where u=30. The highest value is in bold.

Models	Amazon Beauty		Amazon Games		Steam		ML-1M		ML-10M	
	MAP	NDCG	MAP	NDCG	MAP	NDCG	MAP	NDCG	MAP	NDCG
ATAE-LSTM	0.341	0.352	0.421	0.416	0.351	0.425	0.268	0.426	0.435	0.512
IAN	0.335	0.356	0.426	0.419	0.358	0.418	0.262	0.418	0.428	0.435
BILSTM-ATT-G	0.346	0.348	0.418	0.426	0.362	0.408	0.295	0.423	0.426	0.446
MemNet	0.335	0.356	0.425	0.423	0.368	0.415	0.286	0.419	0.438	0.438
TNet	0.339	0.354	0.431	0.428	0.345	0.421	0.297	0.431	0.432	0.446
Ours	**0.352**	**0.362**	**0.435**	**0.441**	**0.416**	**0.432**	**0.325**	**0.438**	**0.443**	**0.536**
